# Analysis and identification of ferroptosis-related diagnostic markers in rheumatoid arthritis

**DOI:** 10.1080/07853890.2024.2397572

**Published:** 2024-09-02

**Authors:** Yang Cai, Lingchuan Deng, Jun Yao

**Affiliations:** Department of Bone and Joint Surgery, The First Affiliated Hospital of Guangxi Medical University, Nanning, China

**Keywords:** Rheumatoid arthritis, ferroptosis, diagnostic marker, signature gene, targeted therapy

## Abstract

**Background:**

Rheumatoid arthritis (RA) is an autoimmune, inflammatory joint disease. There is growing evidence that ferroptosis is involved in the pathogenesis of RA. This study aimed to search for diagnostic markers of ferroptosis in RA and to analyse the potential mechanisms and clinical value.

**Materials and methods:**

RA-associated datasets were used from the publicly available GEO database. Three methods of machine learning were applied to screen biomarkers. The diagnostic efficacy of the results was also verified by receiver operating characteristic (ROC) curve, external dataset, qRT-PCR and Western blot. Enrichment analysis was performed in this process, while protein–protein interaction (PPI) analysis and immune infiltration correlation analysis were performed using biomarkers, and competing endogenous RNA (ceRNA) networks were constructed to search for prospective therapeutic targets.

**Results:**

MMP13 and GABARAPL1 can be used as ferroptosis diagnostic genes in RA. The ROC curve and PPI result demonstrated that MMP13 and GABARAPL1 had an excellent diagnostic value. The results of signature genes in the external dataset, qRT-PCR and Western blot further confirm our findings. The enrichment analysis showed that p53, MAPK and NOD-like receptor signalling pathways may be involved in the process of ferroptosis in RA. In addition, two ferroptosis diagnostic genes in RA participate in the occurrence of ferroptosis in RA via oxidative stress, metabolism and immune response. Immune infiltration analysis showed that RA extensively infiltrated B cells, T cells, macrophages and other immune cells. Persistent immune activation may be an essential reason for the progression of ferroptosis in RA. We also obtained five potential therapeutic agents for RA and some long non-coding RNAs (lncRNAs) and microRNAs (miRNAs) regulating ferroptosis diagnostic genes.

**Conclusions:**

Our study suggests that MMP13 and GABARAPL1, which are closely linked with oxidative stress and immunological modulation, can be used as ferroptosis-related potential diagnostic markers in RA and provide new clues regarding the diagnostic and therapeutic targets of ferroptosis in RA.

## Introduction

Rheumatoid arthritis (RA) is an autoimmune disease that attacks multiple joints throughout the body and can cause irreversible joint damage and disability [1]. Swelling and inflammation of the joint synovium, destruction of cartilage, production of autoimmune antibodies, and systemic multi-organ lesions are the main pathological features of RA [2]. Nonsteroidal anti-inflammatory medications, disease-modifying antirheumatic drugs and glucocorticoids are among the medications used, which can reduce the symptoms or slow the course of RA. However, their complex pathogenic mechanisms and adverse drug reactions vastly limit the implementation of treatment options [3]. These medications cannot alter the nature of RA; they can only partially ease the patient’s symptoms. Current evidence suggests that ferroptosis in fibroblast-like synoviocytes (FLSs) and synovial giant cells is associated with RA development, and ferroptosis also triggers chondrocyte destruction in RA [4,5]. Therefore, finding the diagnostic markers that precisely regulate ferroptosis in RA is of great clinical importance in guiding the therapeutic strategy for RA.

Mitochondrial structure and function abnormalities are the primary morphological hallmarks of ferroptosis, a regulated cell death [6]. The pathomechanism is manifested by increased reactive oxygen species (ROS) due to lipid peroxidation and massive intracellular iron deposition, ultimately leading to cellular dysfunction [7]. Many studies have shown that releasing intra-articular proinflammatory factors in RA patients promotes iron deposition in the synovium, maintaining a persistent synovial inflammatory state [8]. ROS and the severity of RA are correlated favourably. In recent years, the development of high-throughput sequencing has allowed us to recognize the development process of disease from multiple dimensions, such as somatic mutations and altered genomic expression [9,10]. It was found that specific ferroptosis-related genes (FRGs) are involved in the progression of RA. For example, Xiang et al. found that the FRG, SLC2A3, had elevated expression in RA tissues by analysis and identification, and showed down-regulation in response to ferroptosis inducers (RSL3), thus causing the occurrence of ferroptosis in RA [11]. As an FRG, RRM2 has also been found to be differentially expressed between RA patients and normal subjects, and the viability of damaged cells is also increased with the inhibition of this gene, it is thought that FoxO signalling pathway and inherited metabolic disorders play a role in this [12]. VEGFA, PTGS2 and JUN are also thought to be involved in the regulation of ferroptosis in RA by interacting with immune cells [13]. This implies that a few critical genes play a crucial role in the ferroptosis of RA. Ferroptosis has received increasing attention in the field of RA research in recent years. Exploring the relationship between ferroptosis and RA at the level of gene targets and identifying corresponding therapeutic strategies seem to be an up-and-coming area.

Some studies have explored ferroptosis diagnostic markers in RA, but fewer studies have validated their diagnostic value. Based on bioinformatics, ferroptosis signature genes (FSGs) of RA were identified by machine learning and experimental validation methods. In this study, we first obtained the differentially expressed FSGs between RA and normal samples by three datasets, GSE29746, GSE55235 and GSE55457, and validated their diagnostic value using the external dataset GSE77298. Then, we further validated the results obtained based on the GEO dataset by qRT-PCR and Western blot *in vitro* experiments. The study will help explore the potential biological targets associated with ferroptosis in RA, analyse their regulatory mechanisms, and provide a different strategy for treating RA.

## Materials and methods

### Data acquisition and pre-processing

Four RA-related mRNA transcriptome datasets (GSE29746, GSE55235, GSE55457 and GSE77298) were downloaded from the GEO (https://www.ncbi.nlm.nih.gov/geo/) database. The dataset was processed using the R package as follows: GSE29746, GSE55235 and GSE5545 were corrected using the ‘limma’ and ‘SVA’ R packages to remove batch effects [14]. Then, log2 processing is performed on the data with large values, and finally, the data are merged. All the combined data were analysed with relative logarithmic expression (RLE) using R software, and the results were visualized with boxplot (Supplementary Figure 1). The training group included 32 RA synovial samples and 30 normal synovial samples. In addition, GSE77298 were used as independent sequences as the validation group, including RA/normal synovium samples, were 16/7, respectively. Eight hundred and forty-four FRGs were downloaded from the ferrdb database (http://www.zhounan.org/ferrdb).

### Acquisition of ferroptosis differentially expressed genes (DEGs)

The expression of FRGs was extracted from the training group using the ‘limma’ R package. DEGs for ferroptosis between RA and normal synovial samples were then extracted using the ‘limma’ R package as defined by *p* values <.05, and the results were visualized using volcano plot and heat maps. Correlation analysis of DEGs was performed using the ‘corrplot’ R package.

### Enrichment analysis

Correlation analysis of DEGs’ biological functions and signalling pathways was conducted using the clusterProfiler ‘R’ package [15]. The process used Gene Ontology (GO) and the Kyoto Encyclopedia of Genes and Genomes (KEGG). GO consists of three components, biological processes (BPs), cellular components (CCs) and molecular functions (MFs). *p* < .05 was used as the setting condition.

### Candidate FSGs selection

We used three machine learning algorithms to screen for reliable FSGs in RA. LASSO regression analysis was performed to select variables by a regularized analysis algorithm to identify genes that significantly differed between RA synovium and normal synovium. The SVM-RFE algorithm selects and visualizes the most relevant FSGs through a non-linear kernel and accurately interprets the direction and strength of the association between the FSGs and the results [16]. WGCNA can find the most relevant gene modules for the disease by a weighted approach [17]. The ‘glmnet,’ ‘e1071R,’ and ‘WGCNA’ R packages were separately used for analysis to recognize the genes that are of diagnostic significance. The genes obtained from the three methods were crossed to get FSGs for subsequent analysis and validation.

### Confirming the reliability of RA FSGs for diagnosis and gene set enrichment analysis (GSEA)

After obtaining the FSGs crossed by the three algorithms, the receiver operating characteristic (ROC) curves of the FSGs were plotted using the pROC R package, and their diagnostic value was assessed based on the area size of the ROC curves. Meanwhile, to verify the accuracy of these FSGs as ferroptosis diagnostic genes for RA, we used an independent dataset, GSE77298, to validate the expression differences of the FSGs. Finally, we performed GSEA analysis on the FSGs.

### Construction of PPI network of FSGs

To explore the intrinsic association of FSGs with FRGs and pathways, a PPI network with medium confidence (minimum required interaction score of 0.4) was obtained using the STRING database to elucidate the potential mechanism of action.

### Immune infiltration correlation analysis

The differences in the relative abundance of 22 infiltrated immune cells in normal and RA synovial samples from the training group were analysed by the CIBERSORT algorithm [18]. Spearman’s correlation analysis was performed on the infiltration of immune cells and FSGs. Vioplot and ggplot2 R packages plot violin plots and correlation heat maps to analyse and visualize the results.

### Prediction of potential drugs for RA and construction of competing endogenous RNA (ceRNA) network

Based on FSGs, we identified potential therapeutic agents for RA by using the gene–drug interaction database (DGIdb) (https://www.dgidb.org/) [19]. To explore the interactions between FSGs and post-transcriptional regulators long non-coding RNA (lncRNA) and microRNA (miRNA), we constructed a ceRNA network between the three using miRanda, miRDB, TargetScan and SpongeScan bioinformatics tools. The regulatory network was analysed and visualized using Cytoscape_v3.9.1 software [20].

### Extraction and cultivation of synovial cells

In this study, five pairs of RA and normal synovial tissue were collected from the First Affiliated Hospital of Guangxi Medical University (Nanning, China) between June 2023 and September 2023. Synovial tissue for RA was obtained from five patients who fully consented to knee arthroplasty and met the diagnostic criteria for ACR [21]. The control group was from five patients who required arthroscopic surgical treatment for anterior cruciate ligament injury or meniscal injury. The synovial membrane was taken from the normal area within the joint. All patients were hospitalized in the First Affiliated Hospital of Guangxi Medical University. Written informed consent of each patient was obtained during the sample extraction process. Detailed clinical details are provided in Supplementary Table 1. Briefly, synovial tissue was rinsed 2–3 times with phosphate-buffered saline (PBS) and then well minced and digested in DMEM medium (Gibco, Pleasantville, NY) containing type I collagenase (1 mg/mL; Gibco, Pleasantville, NY) for 4–6 h. Next, synovial cells were filtered using a strainer and centrifuged to obtain synovial cells. The cells were cultured in a DMEM medium containing FBS (20%, Gibco, Pleasantville, NY) and penicillin/streptomycin (1%, Solarbio, Beijing, China) in an incubator at 37 °C with 5% CO_2_. The experimental cells were third-generation synovial cells. All procedures of this experiment were approved by the Ethics Committee of the First Affiliated Hospital of Guangxi Medical University (approval number: 2023-E206-01).

### RNA extraction and qRT-PCR

RNA was extracted from RA and normal samples according to the instructions of the RNeasy™ Plus Animal RNA Extraction Kit (Beyotime, R0032, Shanghai, China), and 1 μL of the sample was tested for the concentration and purity of the extracted RNA by UV spectrophotometry. A total of 1 mg of RNA was then reverse transcribed to cDNA in a total volume of 20 μL by a reverse transcription tool (Takara, Beijing, China). Real-time qPCR was then performed according to the specifications of SYBR-Green (Thermo Fisher Scientific, Waltham, MA) operating instructions. The 2^−ΔΔCT^ method was used to analyse, and the gene GAPDH was used as a standardized measure. Independent replicate experiments were performed three times, and each set of samples was repeated thrice. Primer sequences are shown in Table 1.

**Table 1.** Primer information on relevant genes in the study.

Gene name	Forward primer (5′ to 3′)	Reverse primer (5′ to 3′)
GAPDH	ACCCACTCCTCCACCTTTGAC	TCCACCACCCTGTTGCTGTAG
MMP13	TGAAGACCCCAACCCTAAACATCC	TCGGAGACTGGTAATGGCATCAAG
GABARAPL1	ATCCCTCCCACCAGTGCTACC	ATAACACCTTCTGCCCCACTTCTC

### Western blot

Protein was extracted from synovial cells using a configured protein lysis buffer, including phosphatase inhibitor cocktail (ComWin Biotech Co., Ltd., Beijing, China), protease inhibitor (MedChemExpress, Monmouth Junction, NJ), RIPA lysate (Solarbio, Beijing, China) and PMSF (Solarbio, Beijing, China). After the loading buffer was obtained, the protein concentration was determined by BCA protein assay (Beyotime, Shanghai, China). Protein samples were separated by 7.5% and 15% polyacrylamide gels and transferred to polyvinylidene fluoride (PVDF) membranes, then closed with protein-free fast blocking buffer (Shanghai, China) for 30 min. The membranes were soaked in the corresponding primary antibody GAPDH (Proteintech, Shanghai, China), MMP13 (Proteintech, Shanghai, China), GABARAPL1 (Abcam, Cambridge, UK) and incubated overnight in a shaker at 4 °C. Western blot was visualized using ProteinSimple’s Wes system. Gray scale values of Western blot were analysed using Image-J software (BioRad, Hercules, CA).

### Statistical analysis

All bioinformatics analyses were performed on R v.4.1.13. Differences between the two groups were compared using the Wilcoxon test. Correlations between different variables were analysed using Spearman’s correlation analysis. Experimental data from *in vitro* experiments were analysed using the unpaired two-tailed *t*-test method. A statistically significant difference was considered at *p* < .05.

## Results

### DEGs associated with ferroptosis and enrichment analysis

**Figure 1.**
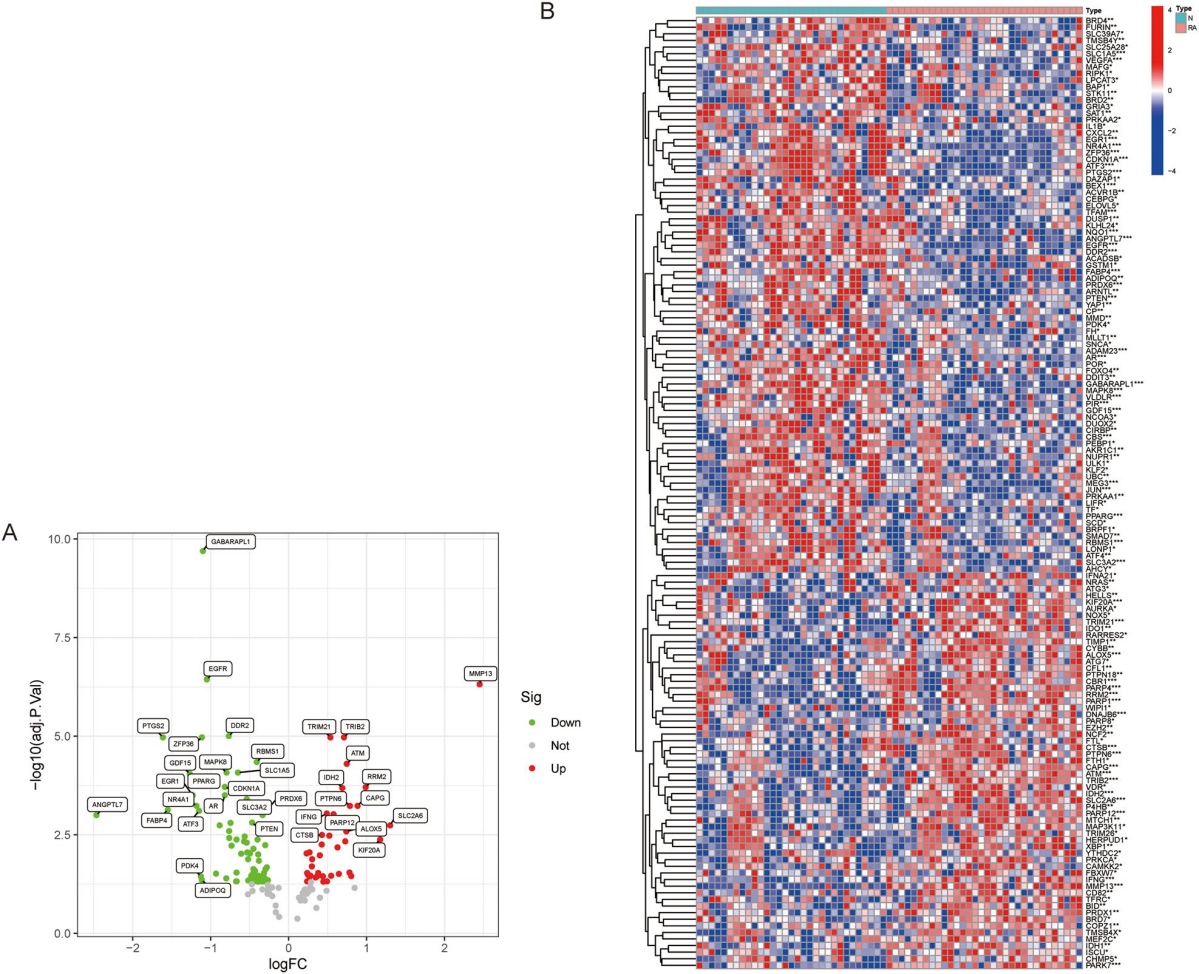
Ferroptosis DEGs between RA and normal samples. (A) Volcano plot of upregulated and down-regulated genes. (B) Heat maps of DEGs, red for up-regulation, blue for down-regulation and white for no difference.

**Figure 2.**
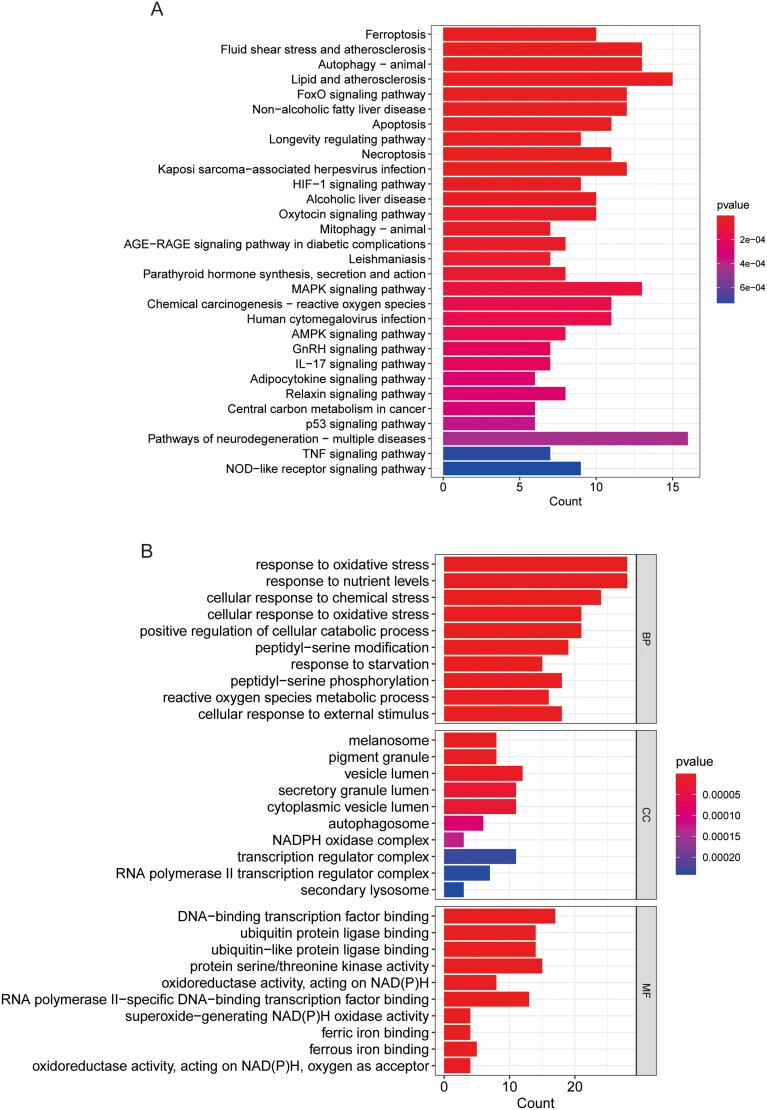
Functional enrichment analysis. (A) KEGG. (B) GO.

We obtained the combined cohort (GSE29746, GSE55235 and GSE5545) after eliminating batch effects using the ‘limma’ and ‘SVA’ R packages. From 844 FRGs downloaded through the ferrdb database, we identified 389 FRGs in the sequence set. We further screened 144 DEGs related to ferroptosis in RA in the training group, of which 60 were upregulated and 84 were down-regulated. Figure 1(A,B) (volcano plot and heat map) shows the distribution of DEGs among different samples. We used KEGG and GO enrichment analysis to demonstrate the biological functions and signalling pathways associated with the DEGs. KEGG showed the relationship between DEGs and MAPK/NOD-like receptor (NLR) signalling pathway, p53 signalling pathway, ferroptosis and apoptosis (Figure 2(A)). Elevated expression of P53 inhibits SLC7A1 1/GPX4 expression and thus induces cellular ferroptosis [22]. Ferroptosis is a cell death regulated by intracellular iron deposition and lipid peroxidation and is closely related to the intracellular antioxidant system. In GO (Figure 2(B)), DEGs were significantly enriched in cellular response to oxidative stress, ROS metabolic process, positive regulation of cellular catabolic process, and other BPs, indicating that DEGs are closely associated with inflammatory response processes related to iron and lipid metabolism. In CC, DEGs were enriched in autophagosome, NADPH oxidase complex, cytoplasmic vesicle lumen and secretory granule lumen. In MF, DEGs were associated with oxidoreductase activity, superoxide-generating NAD(P)H oxidase activity, iron/ferrous binding and protein serine/threonine kinase activity, suggesting that DEGs are involved in processes of cellular iron metabolism and antioxidant regulation. The key to ferroptosis lies in intracellular iron deposition and the weakening of lipid antioxidant function. Enhancing the activity of antioxidant system and inhibiting iron deposition are effective measures to treat ferroptosis in RA [23].

### Screening and identification of FSGs

**Figure 3.**
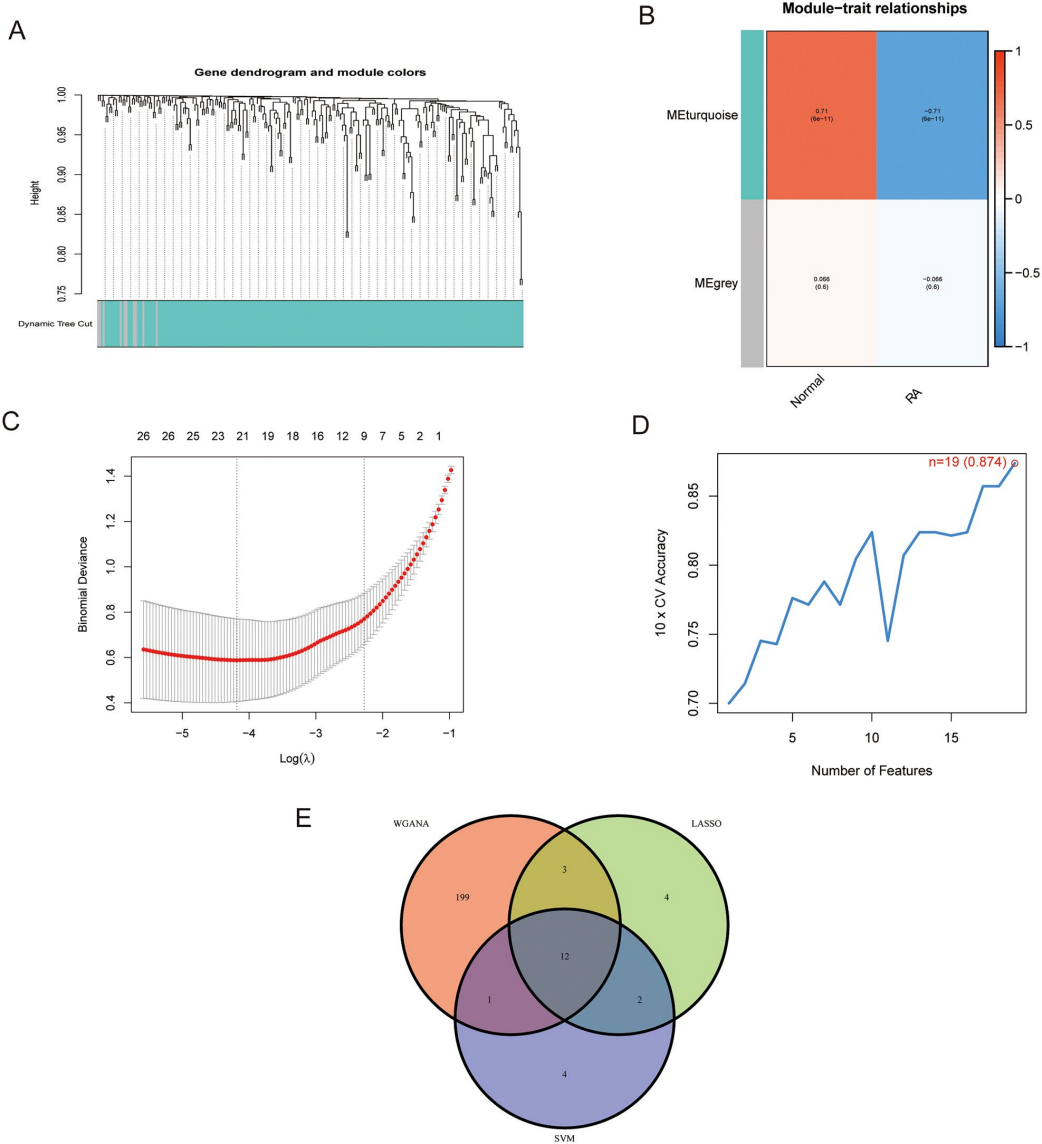
Screening of RA FSGs. (A) Dendrogram of WGCNA gene modules. The top and bottom corresponding parts are the hierarchical clustering map of genes and the gene module map. (B) Module-trait correlation heat map. Each row represents a module and each column represents a trait. The middle two columns represent traits of RA synovium and normal synovium. Blue is a negative correlation, orange is a positive correlation, and the darker the colour, the stronger the correlation. The *p* values of correlation and significance are labelled in the cells. (C) FSGs screened from DEGs using the LASSO regression algorithm. (D) FSGs screened from DEGs using the SVM-RFE algorithm. (E) Venn diagram of intersecting genes obtained by the three methods (WGCNA, LASSO and SVM-RFE).

**Figure 4.**
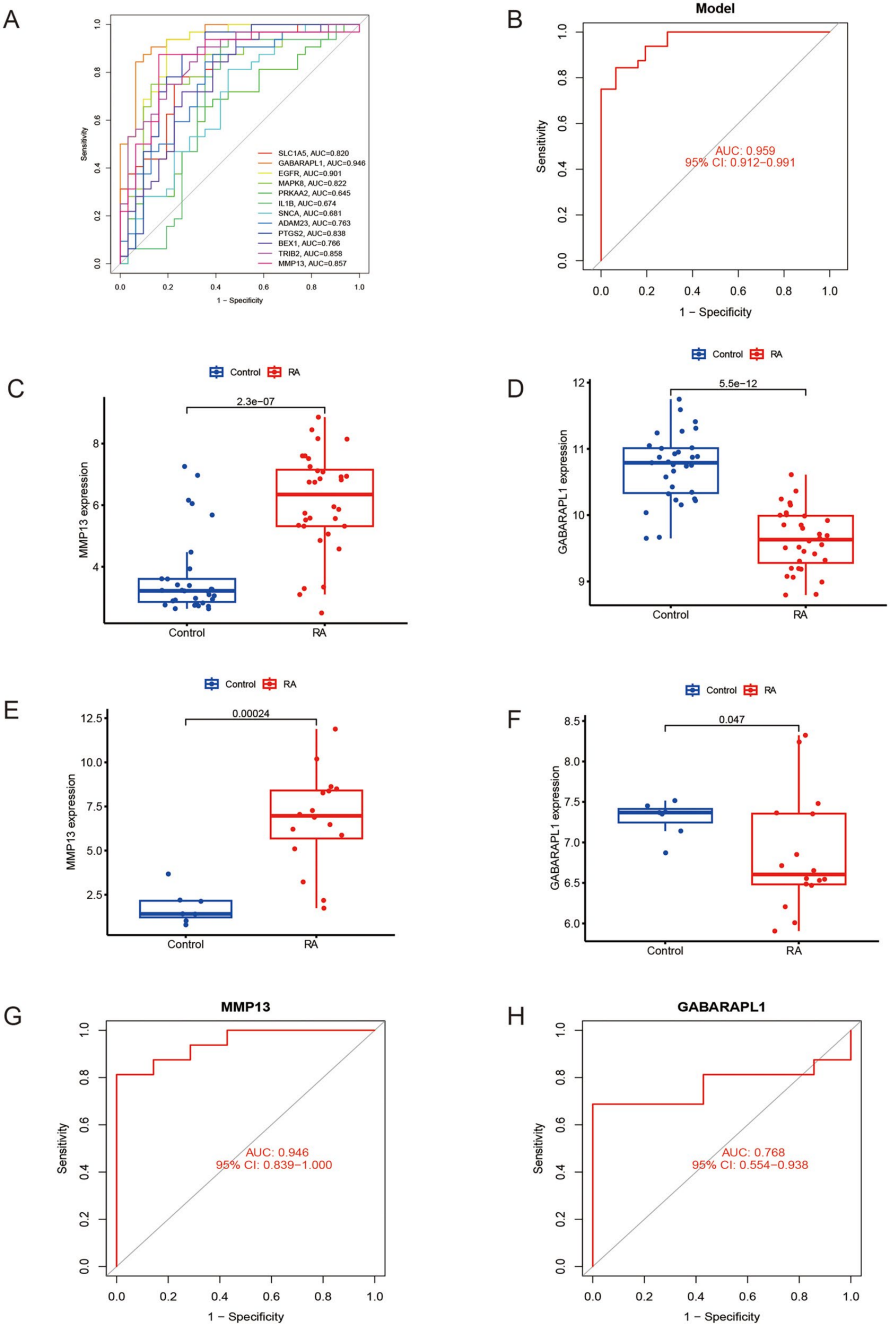
Data set validation and diagnostic value analysis of RA FSGs. (A) ROC curves to validate the diagnostic accuracy of RA’s FSGs. (B) ROC curves of the FSGs (GABARAPL1, MMP13) model. (C, D) Expression of the FSGs in the merged dataset. (E, F) Expression of FSGs in the GSE77298 dataset. (G, H) ROC curves of the FSGs in the GSE77298 validation group.

We first screened 389 FRGs in the training group using the WGCNA analysis method. Different modules were identified according to the correlation between different gene-traits (Figure 3(A)). The absolute value of correlation was taken and the module gene with the highest absolute value had the highest correlation with RA. Therefore, the MEturquoise gene module, which includes 215 ferroptosis-associated genes, was considered (Figure 3(B)). Using the LASSO logistic regression algorithm, we screened 21 DEGs from the 144 DEGs related to ferroptosis in RA (Figure 3(C)). Similarly, we screened out 20 DEGs using the SVM-RFE algorithm (Figure 3(D)). Finally, the genes obtained from the above three methods were crossed to get 12 FSGs (SLC1A5, GABARAPL1, EGFR, MAPK8, PRKAA2, IL1B, SNCA, ADAM23, PTGS2, BEX1, TRIB2 and MMP13) (Figure 3(E)). Following that, we constructed ROC curves to assess the diagnostic value of these FSGs and screened them according to the criteria of excellence defined by AUC value ≥0.85 [24]. The results showed that GABARAPL1, EGFR, TRIB2 and MMP13 met the inclusion criteria, with AUC values of 0.946, 0.901, 0.858 and 0.857, respectively (Figure 4(A)). We next validated the 4 FSGs using the external dataset GSE77298 (*p* < .05 was deemed significant). The results showed that only two FSGs, MMP13 and GABARAPL1, passed the validation and were consistent with the expression trend of the training group (Figure 4(C–F)). We also constructed model ROC curves for these two FSGs with an AUC value of 0.959 (Figure 4(B)). We also examined the diagnostic accuracy of these two FSGs in the GSE77298 cohorts. The AUC values of MMP13 and GABARAPL1 were 0.946 and 0.768, respectively (Figure 4(G,H)). The above results showed that these two FSGs provided good diagnostic accuracy.

### Co-expression network of FSGs and ferroptosis-related proteins

**Figure 5.**
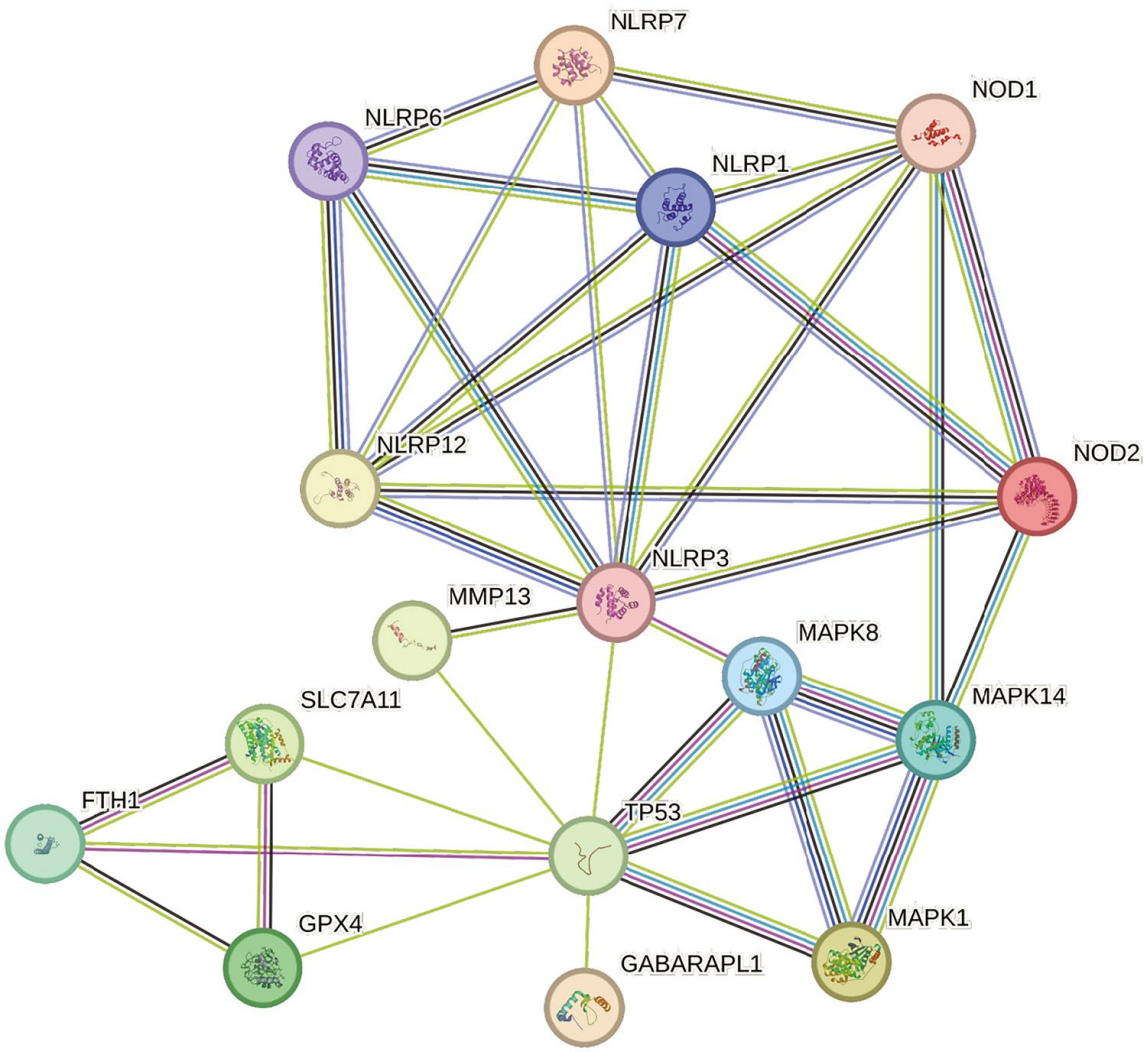
Exploring the relationship between FSGs and potential signalling pathways of RA or FRGs.

To elucidate the potential relationship between the FSGs and ferroptosis-related proteins, we constructed a PPI network diagram between two FSGs, ferroptosis-related proteins (FTH1, GPX4, SLC7A11 and P53) and related pathways through the STRING database (Figure 5). We found a close interaction between the FSGs and ferroptosis-related proteins.

### Gene set enrichment analysis

**Figure 6.**
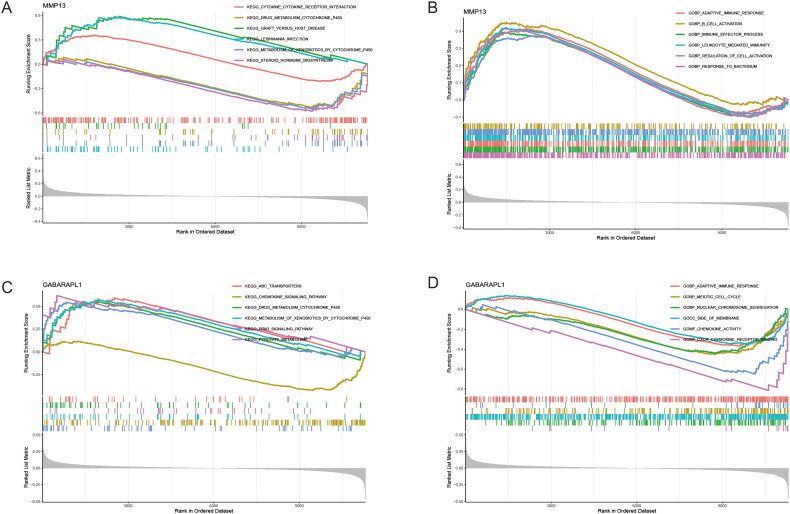
GSEA of the FSGs. (A, B) KEGG and GO in MMP13. (C, D) KEGG and GO in GABARAPL1.

Based on the expression profile of the FSGs, GSEA analysis revealed the functional and pathway enrichment of the FSGs in the high and low expression groups. We found that the up-regulation of MMP13 expression was closely related to the cytokine receptor interaction, graft versus host disease, Leishmania infection, adaptive immune response, B cell activation, immune effector process, leukocyte mediated immunity and regulation of cell activation (Figure 6(A,B)). This suggests that upregulated MMP13 may mediate ferroptosis in RA by activating the body’s immune response. More interestingly, the down-regulation of GABARAPL1 was significantly enriched in chemokine signalling pathway, adaptive immune response, meiotic cell cycle, nuclear chromosome segregation, side of membrane, chemokine activity and CXCR chemokine receptor binding (Figure 6(C,D)). This suggests that FSGs play a role in immune response, cell cycle and metabolism, cytokine interactions, protein synthesis and metabolism. All of these are associated with ferroptosis in RA. Analyses of the expression of FSGs have improved our understanding of the pathogenesis related to ferroptosis in RA.

### Immune cell infiltration

**Figure 7.**
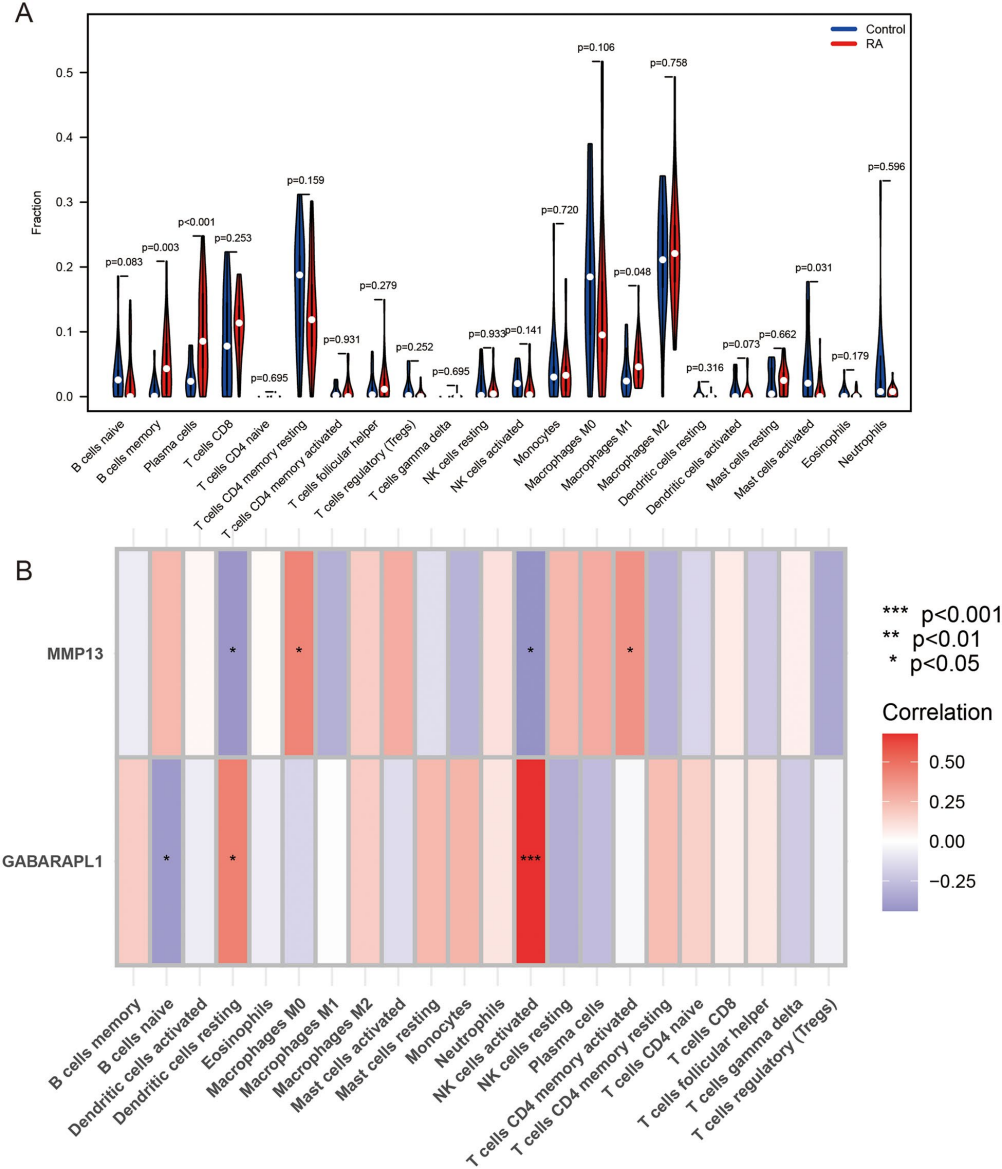
Analysis of immune cell infiltration. (A) The difference of 22 kinds of immune cell infiltration between RA group and normal sample group. (B) Correlation heat maps between 2 FSGs and 22 types of immune cells. Blue means negative correlation, red means positive correlation (*p < .05; **p < .01; ***p < .001).

An autoimmune disease affecting joints throughout the body; RA is distinguished by the infiltration of immune cells and the production of autoimmune antibodies [25]. We found differences in B-cell memory, plasma cells, macrophages M1 and mast cells activated by analysing 22 immune cell infiltrations between RA and normal samples (Figure 7(A)). The expression of most immune cells is upregulated in RA. We also plotted the heat map between FSGs and immune cells. The results showed that FSGs were positively correlated with macrophages M0, T cells CD4 memory activated, dendritic cells resting and NK cells activated, and negatively correlated with dendritic cells resting and B cells naive (Figure 7(B)).

### Signature gene–drug interaction network and ceRNA regulatory network

**Figure 8.**
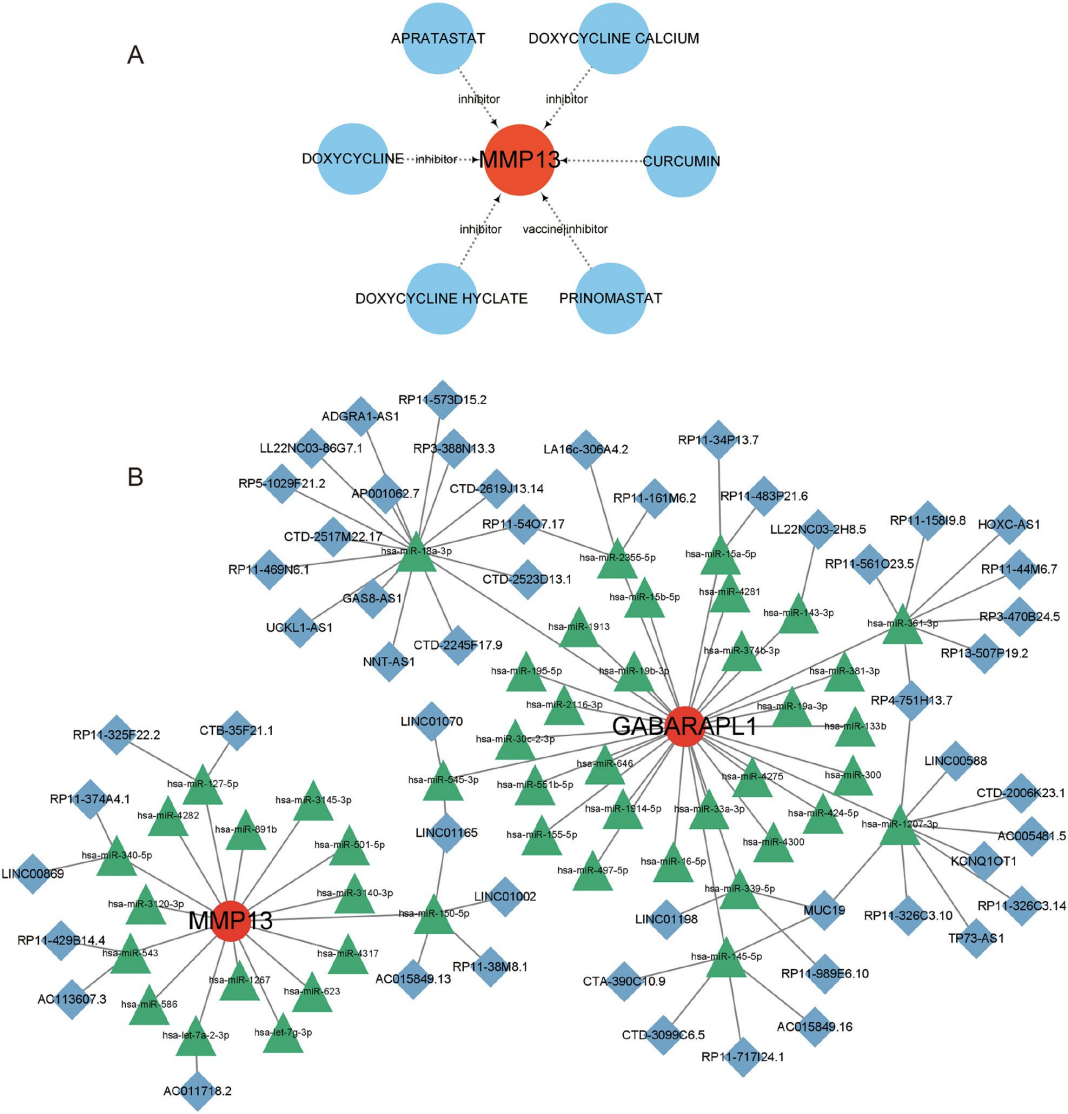
Gene networks of RA potential drugs and ceRNA regulatory networks. (A) The potential drug-FSGs network of RA was constructed by cytoscape, with red representing genes and blue representing drugs. (B) ceRNA network regulation map of lnRNA-miRNA-FSGs.

We used DGIdb to identify five potential therapeutic agents for RA: apratastat, prinomastat, doxycycline hyclate, doxycycline and doxycycline calcium (Figure 8(A)). To observe which FSGs and lncRNAs competitively bind miRNAs, we constructed a ceRNA network among the three and finally obtained the interaction network diagram of two mRNAs, 53 lncRNAs and 47 miRNAs (Figure 8(B)). We found that GABARAPL1 and lncRNA competitively bind has-miR-18a-3p, has-miR-2355-5P, has-miR-15a-5p, has-miR-145-5p, has-miR-361-3p, has-miR-1207-3p and has-miR-339-5p. MMP13 and lncRNA competitively bind to has-miR-127-5p, has-miR-340-5p, has-miR-543 and has-miR-150-5p. This suggests that different lncRNAs and miRNAs can regulate FSGs competitively.

### Identification of the expression levels of two FSGs

**Figure 9.**
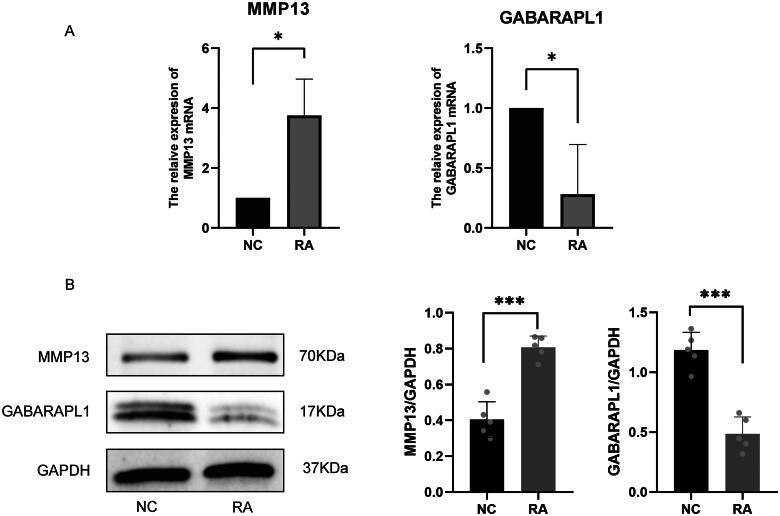
Expression levels of two FSGs. (A) qRT-PCR analysis of MMP13 and GABARAPL1 in normal and RA. (B) Western blot analysis verified that MMP13 and GABARAPL1 protein expression and quantification (means ± SD, *n* = 5, *p < 0.05; **p < 0.01; ***p < 0.001).

In this study, two FSGs were detected by qRT-PCR and Western blot to identify their expression between RA patients and normal subjects. Compared with normal samples, the mRNA expression of MMP13 increased in RA samples, while GABARAPL1 decreased (Figure 9(A)). Western blot measures protein levels of MMP13 and GABARAPL1 in synovial cells. When comparing RA samples to normal samples, higher protein levels of MMP13 and lower protein levels of GABARAPL1 were found in the former (Figure 9(B)).

## Discussion

RA is a long-lasting, inflammatory joint condition. The invasion of synovial inflammation and cartilage destruction is often accompanied by adverse joint results and ultimately leads to disability. As a disease with high activity, the production of autoantibodies and the appearance of early joint damage imply the importance of early diagnosis and intervention. Therefore, the search for useful diagnostic markers and new therapeutic approaches is clinically important for early recognition and intervention of the disease. Many studies have found that ferroptosis, a programmed cell death distinct from apoptosis and characterized by iron deposition and lipid peroxidation, plays a vital role in the pathological process of RA [26,27]. However, the relationship between FRGs and RA and the mechanism of action has not been clarified.

In the study, two FSGs of RA were identified. The findings of immune infiltration analysis and the interactions between lncRNA, miRNA and FSGs have triggered our thinking on the relationship between RA and ferroptosis. RA is currently a disease with no established etiology and pathogenesis. However, pertinent research has demonstrated that alterations in specific pathways and persistent immunological activation are intimately linked to the incidence and progression of RA [28]. Enrichment analysis showed that 144 ferroptosis DEGs were significantly enriched in MAPK/NLR/p53 signalling pathway and involved in iron deposition and oxidation regulation processes. The enrichment result suggests these genes play an important role in RA ferroptosis. Activation of the MAPK inflammatory pathway has been shown to down-regulate the expression of SLC7A11 and GPX4, leading to ferroptosis [29]. Xie et al. found that activation of MAPK pathway contributes to ferroptosis of cancer cells [30]. Meanwhile, Li et al. also found that the ferroptosis pathway mediated by ROS/MAPK and p53 can kill nasopharyngeal carcinoma cells [31]. As a crucial regulator of ferroptosis, the dysfunction of p53 in FLSs is associated with synovitis and the destruction of bone and cartilage. Sun et al. indicated that elevated expression of p53 could induce ferroptosis in cells through the p53/SLC7A1/GPX4 pathway [22]. However, there are few studies on the correlation between the expression of p53 and ferroptosis in RA [32]. As a family of cytoplasmic proteins, the NLRs family plays a crucial role in pathogen recognition and innate immune response. By identifying the appropriate ligand, the NLR signalling pathway can activate the downstream signalling pathway NF-κB and then promote the transcription of the inflammasome NLRP3. Next, NLRP3 induces the activation of caspase-1, which promotes the processing and secretion of precursors of IL-1β and IL-18 [33]. A crucial ferroptosis regulator is GPX4. In RA, the overactivation of NLRP3 may limit the production of GPX4, resulting in a significant buildup of ROS and iron-dependent cell death [34]. Several pathways, including MAPK and apoptosis, are stimulated by the NLR signalling pathway at the same time [35]. The activation of a series of pathways and the release of inflammatory factors initiate the immune response process. We built a PPI network to investigate the connections between FSGs, ferroptosis-related proteins and associated pathways. The findings suggest that FSGs may have a role in ferroptosis directly or indirectly via related pathways, but the precise mechanism remains to be further investigated and discussed.

MMP13 is the protease that plays a major role in RA and is produced by chondrocytes [36]. Synovitis of RA releases inflammatory cytokines that stimulate MMPs production. MMPs can degrade collagen and aggrecan of the extracellular matrix of the cartilage, thereby causing irreversible destruction of the articular cartilage, bone and tendon. Cartilage fragmentation in turn irritates synovium, leading to aggravation of inflammation [37]. The buildup of ROC also can activate MMP13. The elevated expression of MMP13 can cause more severe joint damage in RA patients [38]. By interacting with the NF-B binding site on the MMP gene, the transcription factor AP-1 can increase the expression of MMP [39]. This suggests that MMP13 may be related to the NLR signalling pathway and involved in the process of ferroptosis. However, the mechanism of MMP13 in ferroptosis in RA is unclear and was screened as FSG in this study, which piqued our curiosity. GABARAPL1 belongs to one of the subfamilies of the yeast autophagy-related eight families, which has the function of transporting vesicles and proteins, regulating energy metabolism and oxygen radical homeostasis, and is involved in various pathological processes such as autophagy, cell proliferation and death [40]. It has promise as a molecular target for autoimmune or inflammatory diseases [41]. Autophagy, particularly selective ferritinophagy, increases lipid peroxidation and iron accumulation, resulting in cellular ferroptosis [42]. Down-regulation of GABARAPL1 in hepatocellular carcinoma cells diminished their sensitivity to erastin, a ferroptosis inducer, according to Du et al. [43]. Interestingly, down-regulated GABARAPL1 expression was also found in whole blood of RA patients [44]. We believe that GABARAPL1-mediated autophagy can cause cells to undergo ferroptosis. The role of these two FSGs for ferroptosis in RA remained unclear and was identified for the first time as diagnostic markers of ferroptosis in our study. The diagnostic value of these two FSGs was also demonstrated by ROC analysis. This was also confirmed in our *in vitro* experiments, in which MMP13 showed significant upregulation of expression in RA samples, while GABARAPL1 showed significant downregulation of expression. The above results suggest that these two FSGs can be used as potential ferroptosis-related diagnostic markers for RA.

RA is an autoimmune disease in which the infiltrated immune cells in the joint and the existing autoantibodies participate in the whole process of the disease [45]. In the study, RA samples were shown to have higher B cells memory, plasma cells and macrophages M1 infiltration. FSGs are associated with macrophages M0, T cells CD4 memory activated, dendritic cells resting, NK cells activated and B cells naive. According to studies, the leading causes of joint damage are MMP-1 and MMP-13 released by synovial cells in RA [46]. At the same time, it has been shown in RA that lymphocytes and monocytes promote the expression of MMPs and cytokines utilizing microparticles [47]. According to the available data, the GABARAPL1 protein is crucial for adaptive immunity because it processes and presents antigens via MHC-I and MHC-II molecules, promoting the production of CD8+ or CD4+ T cells [48]. Additionally, the MHC-I protein in dendritic cells uses the autophagy of GABARAPL1 to cross-present antigens [49]. Activation of immune cells such as T cells, B cells, dendritic cells and macrophages promotes the production of rheumatoid factor and ACPA, leading to increased synovial inflammation and irreversible joint destruction [50]. The uncontrolled adaptive immune response and activation of innate immunity will exacerbate the progression of ferroptosis in RA. Various immune cells, mainly B cells, T cells and macrophages, play an essential role in the ferroptosis development of RA, and our study yielded consistent results [45]. We also identified five potential therapeutic agents, including apratastat, prinomastat, doxycycline hyclate, doxycycline and doxycycline calcium. Moreover, to determine which lncRNAs compete with FSGs for miRNA binding, we obtained an interrelationship network of two mRNAs, 53 lncRNAs and 47 miRNAs. Previous studies have indicated that has-miR-19a can reduce the severity of RA through negative regulation of MMP13 [51]. The negative regulation of GABARAPL1 by has-miR-19a and has-miR-145 affected the proliferation and differentiation of mesenchymal stem cells and endothelial progenitor cells [52,53]. These findings indicate the potential for new ferroptosis therapeutic targets in RA.

Therefore, we believe that MMP13 and GABARAPL1 engage in the process of ferroptosis in RA and can serve as valuable diagnostic markers. Besides, they have a close relationship with pathways related to ferroptosis with further potential as therapeutic targets. However, more studies are necessary to explain the interaction between diagnostic markers and ferroptosis. Admittedly, we have to acknowledge the shortcomings of this study. The sample size for analysis and verification is small in the first place. Second, there is a need for further studies to explore the detailed mechanism of the diagnostic markers in RA ferroptosis.

## Conclusions

Through bioinformatics and *in vitro* experiments, two FSGs, MMP13 and GABARAPL1, were confirmed to be differentially expressed between RA and normal patients and could serve as valuable diagnostic biomarkers. Persistent immune activation and excessive oxidative stress as well as the regulation of ferroptosis-related pathways may be the possible mechanisms of ferroptosis caused by diagnostic biomarkers in RA. The lncRNAs and miRNAs associated with diagnostic markers also provide new molecular targets for the treatment of RA.

## Supplementary Material

Supplemental Material

## Data Availability

The original public dataset used in this paper is available for download from the Gene Expression Omnibus (GEO, https://www.ncbi.nlm.nih.gov/geo/) platform. Additional data used to support the results of this study are available from the corresponding author upon request.
