# Supplementary figures and images for: Analysis and identification of ferroptosis-related diagnostic markers in rheumatoid arthritis
Source: Ann Med. 2024 Sep 2;56(1):2397572. doi: 10.1080/07853890.2024.2397572 (PMC11370691; doi:10.1080/07853890.2024.2397572)

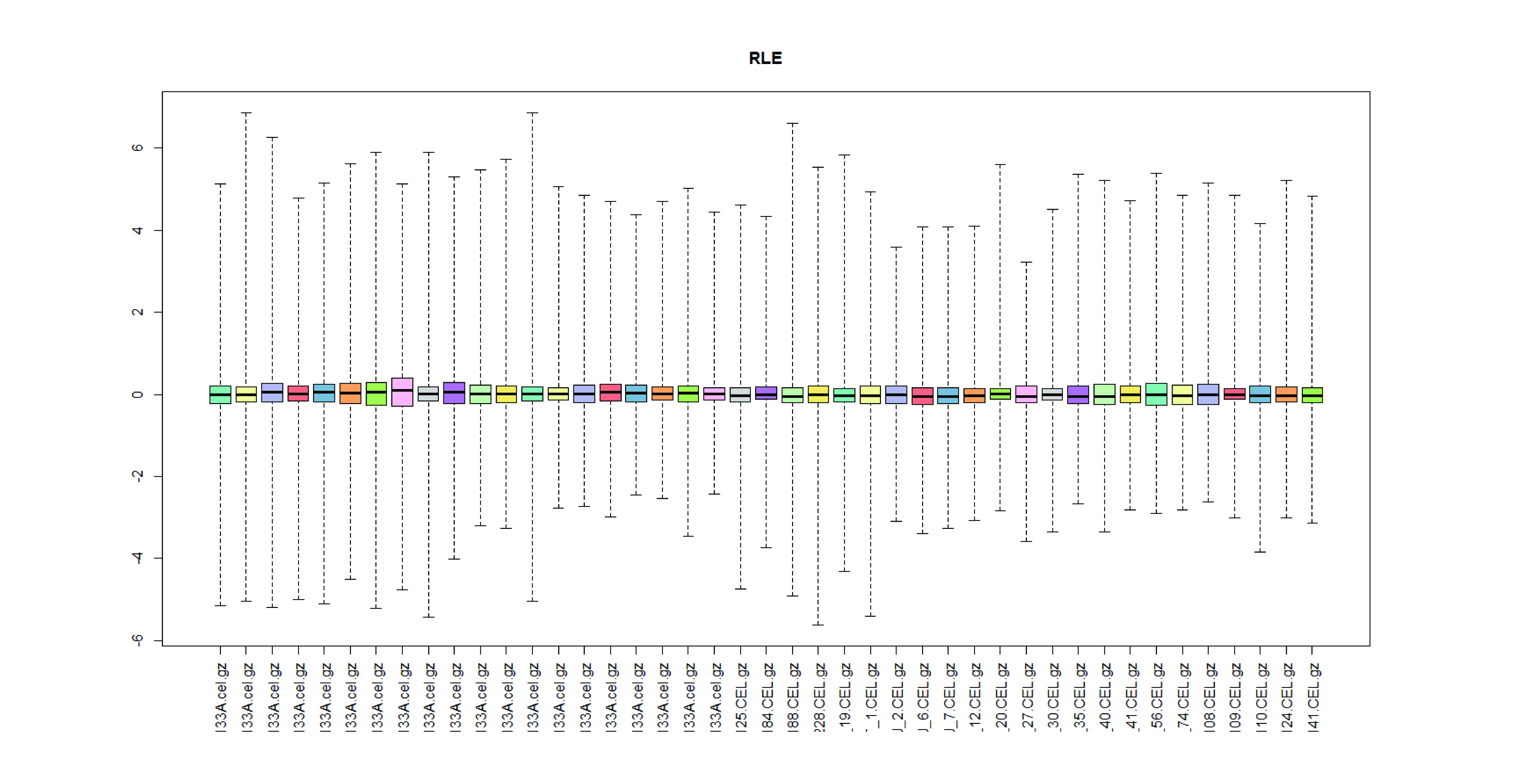

Supplement: Supplemental Material [file IANN_A_2397572_SM9175.zip › Suppl_Data/Supplementary_figure_1.tif]
