# Supplementary material for: Analysis and identification of ferroptosis-related diagnostic markers in rheumatoid arthritis
Source: Ann Med. 2024 Sep 2;56(1):2397572. doi: 10.1080/07853890.2024.2397572 (PMC11370691; doi:10.1080/07853890.2024.2397572)
Supplement: Supplemental Material [file IANN_A_2397572_SM9175.zip › Suppl_Data/Supplementary table 1 (1).doc]

**The detailed data about RA/Non-RA patient**

All the 10 patients in this study were enrolled from the Department of Bone and Joint Surgery, The First Affiliated Hospital of Guangxi Medical University (Nanning, China). Five patients who met the diagnostic criteria for ACR and five patients with anterior cruciate ligament injuries or meniscal injuries were included. Data from a medical history and physical examination were compiled for each patient. The information and histological diagnoses of selected patients were listed in the following table.

**Supplementary Table 1.** Clinical information of the patient

| Number | Gender | Age  (years) | duration  (years/months/days) | RA/Non-RA |
| --- | --- | --- | --- | --- |
| 1 | Female | 56 | 5y | RA |
| 2 | Female | 76 | 30y | RA |
| 3 | Male | 53 | 1y | RA |
| 4 | Female | 46 | 4y | RA |
| 5 | Female | 58 | 8y | RA |
| 6 | Male | 25 | 14d | Non-RA |
| 7  8  9  10 | Male  Male  Female  Female | 33  27  24  31 | 2m  10d  3m  3d | Non-RA  Non-RA  Non-RA  Non-RA |

**Supplementary figure Caption**

**Supplementary figure 1.** The boxplot of combined data was analyzed with relative logarithmic expression (RLE).
